# Golgi protein 73 and its diagnostic value in liver diseases

**DOI:** 10.1111/cpr.12538

**Published:** 2018-10-19

**Authors:** Yanyan Xia, Yuanying Zhang, Mengjiao Shen, Hongpan Xu, Zhiyang Li, Nongyue He

**Affiliations:** ^1^ Department of Clinical Laboratory The Affiliated Drum Tower Hospital of Nanjing University Medical School Nanjing China; ^2^ Department of Molecular Biology Jiangsu Cancer Hospital Nanjing China; ^3^ Center of Laboratory Medicine The Second Affiliated Hospital of Nanjing Medical University Nanjing China; ^4^ State Key Laboratory of Bioelectronics Southeast University Nanjing China

**Keywords:** GP73, hepatitis, liver cancer, liver cirrhosis

## Abstract

Golgi protein 73 (GP73, also referred to as Golph 2) with 400 amino acids is a 73 kDa transmembrane glycoprotein typically found in the cis‐Golg complex. It is primarily expressed in epithelial cells, which has been found upregulated in hepatocytes in patients suffering from both viral and non‐viral liver diseases. GP73 has drawn increasing attention for its potential application in the diagnosis of liver diseases such as hepatitis, liver cirrhosis and liver cancer. Herein, we reviewed the discovery history of GP73 and summarized studies by many groups around the world, aiming at understanding its structure, expression, function, detection methods and the relationship between GP73 and liver diseases in various settings.

## 1 INTRODUCTION

Golgi protein 73 (GP73) is a transmembrane protein that has been found on the Golgi apparatus in recent years. It is also known as Golgi membrane protein I or Golgi phosphor protein 2. Because its relative molecular mass in polyacrylamide gel electrophoresis is 7.3 × 10^4^, it is also called GP73.^1^, ^2^, ^3^, ^4^, ^5^, ^6^, ^7^, ^8^ At present, many studies have found that the GP73 protein is associated with liver diseases, especially with liver cancer diseases^5^, ^9^, ^10^, ^11^, ^12^, ^13^, ^14^, ^15^, ^16^, ^17^, ^18^, ^19^, ^20^, ^21^, ^22^, ^23^; therefore, it is expected to become a new serological tumour marker for early diagnosis of liver cancer, which has attracted widespread attention from research scholars.

The Golgi apparatus is mainly involved in protein secretion, glycosylation and membrane transformation. The maintenance of cellular spatial structure and execution of its functions is closely related to the Golgi's membrane‐resident protein.^20^, ^24^, ^25^, ^26^, ^27^, ^28^, ^29^, ^30^, ^31^, ^32^, ^33^, ^34^, ^35^, ^36^, ^37^, ^38^, ^39^, ^40^, ^41^, ^42^, ^43^ The Golgi apparatus can participate in signal transmission and cell differentiation and can also play an important role in cell apoptosis. Therefore, some functions of Golgi apparatus are abnormal, in particular, abnormal function of certain membrane proteins are most likely related to the occurrence and development of tumours.^44^, ^45^, ^46^, ^47^, ^48^, ^49^, ^50^, ^51^, ^52^

## 2 BASIC OVERVIEW OF GP73

### 2.1 The discovery of GP73

American scholar Kladney et al performed subtractive hybridization in liver cDNA libraries of healthy individuals and patients with acute adult giant cell hepatitis in 2000 and found that the mRNA expression in patients with adult giant cell hepatitis was specifically increased.^53^, ^54^, ^55^, ^56^, ^57^, ^58^, ^59^, ^60^, ^61^, ^62^, ^63^ The mRNA encodes a protein GP73 containing 401 amino acids, and its relative molecular mass is 4.5 × 10^4^.^64^, ^65^, ^66^, ^67^, ^68^, ^69^, ^70^, ^71^ However, due to acidic amino acid effect and glycosylation of GP73, denaturation of GP73 protein was affected, and electrophoretic migration rate was decreased. The relative molecular mass of GP73 in the polyacrylamide gel electrophoresis was slightly larger, GP73 band abnormally migrated in the Western blot, and its relative molecular mass was 7.3 × 10^4^.^72^, ^73^, ^74^, ^75^, ^76^, ^77^, ^78^, ^79^

### 2.2 Structural features of GP73

**Figure 1:**
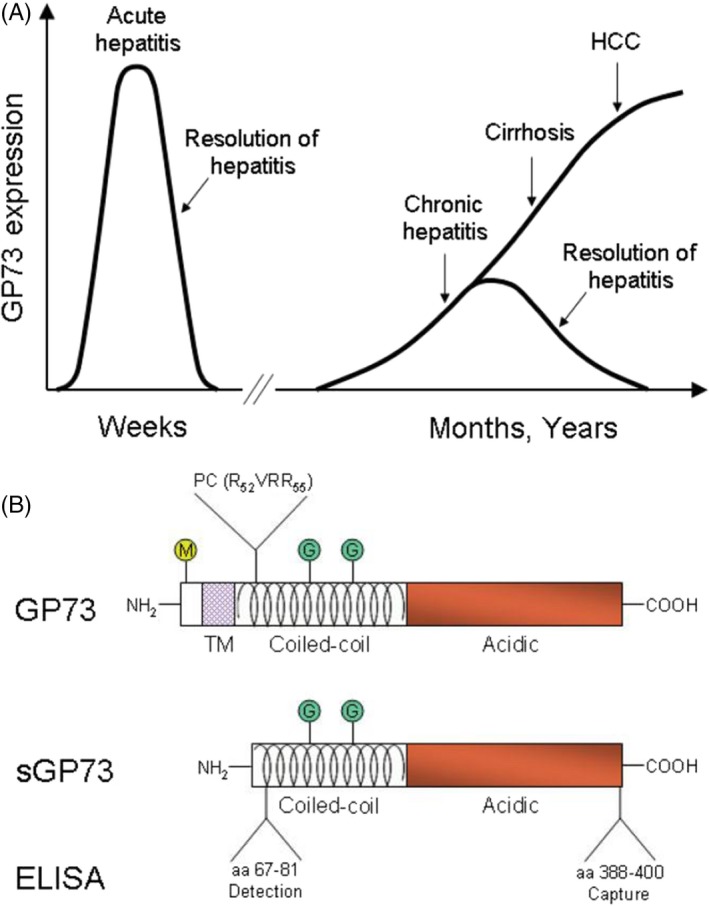
A, GP73 expression levels in hepatocytes in the course of acute and chronic liver disease. Normal hepatocyte GP73 expression is minimal. Marked and reversible increases in the percentage of GP73‐positive hepatocytes, and their cellular GP73 levels occur in acute hepatitis. Progressive up‐regulation is also observed in chronic liver disease and can be reversed by treatment of underlying disease aetiology (ie, steroid treatment of autoimmune hepatitis) and resolution of chronic hepatitis. GP73 expression in hepatocellular cancer cells is further increased (2‐ to 3‐fold) in comparison with patients with cirrhosis. B, Structural features of GP73 and sGP73. GP73 consists of a short cytoplasmic N‐terminus with a myristoylation domain followed by a single TM and large C‐terminal ectodomain. The N‐terminus contains two coiled‐coil domains and is N‐glycosylated. The C‐terminus is highly acidic of unknown function. Furin cleavage after aa 55 results in the extracellular release of sGP73. aa, amino acid; ELISA, enzyme‐linked immunosorbent assay; GP73, Golgi protein 73; HCC, hepatocellular carcinoma; sGP73, serum form of Golgi protein 73; TM, transmembrane domain^103^

The GP73 gene is located in the short arm of chromosome 9 at position 9q21.33. It has a total length of 3042 bp, containing 9 introns and 10 exons. It also contains a unique open‐reading frame (1200‐1430 bp) that encodes 400 amino acids.^80^, ^81^, ^82^, ^83^, ^84^ The 3′ untranslated region of GP73 gene contains three polyadenylation sites (3003, 2950 and 1448 bp) and a stop codon (bp 1351). The encoded protein contains more acidic amino acids, such as aspartic acid and glutamic acid, and the isoelectric point for the amino acid is 4.72, containing single transmembrane region and signal peptidase cleavage site aa28‐aa29 and N‐terminal hydrophobic (Figure 1).^85^, ^86^, ^87^, ^88^, ^89^, ^90^, ^91^, ^92^, ^93^ The amino acid encoded by C‐terminus is located in the extracellular region and contains 5 glycosylation sites and 14 cellulose acylated sequences.^94^, ^95^, ^96^ Several coiled coils immediately after the transmembrane region can participate in the interaction between proteins, and their characteristics indicate that the GP73 protein can interact with other proteins through the extracellular region.

### 2.3 Expression and distribution of GP73

The GP73 promoter −2618 bp/−19 bp has specificity for epithelial cells, so GP73 is widely expressed in almost all epithelial cells.^14^, ^95^, ^97^, ^98^, ^99^, ^100^, ^101^, ^102^, ^103^ Results from immunohistochemistry have also shown that the GP73 protein can be expressed in many types of cells in normal human tissues, but most important is epithelial cells, where the expression levels are very different. The difference may be up to 20 times.^9^, ^77^, ^104^, ^105^, ^106^, ^107^, ^108^ GP73 mRNA is expressed at higher levels in bronchial, gastric, colonic and prostate tissues, but with lower or no expression in muscle, white blood cells, lymphoid tissues and hearts.^72^, ^109^, ^110^, ^111^, ^112^, ^113^, ^114^, ^115^, ^116^, ^117^, ^118^ The most densely expressed GP73 site is the digestive tract. It is also expressed in bile duct epithelial cells, tubular monolayer epithelial cells, pulmonary bronchioles, islets and hippocampus.

The high expression of GP73 belongs to epithelial cells, and these parts have well‐developed Golgi apparatus whose cells have strong secretory ability.^9^, ^119^, ^120^, ^121^, ^122^, ^123^, ^124^ GP73 is mainly expressed in normal liver tissue by bile duct epithelial cells in the portal area. This site is typical for the Golgi apparatus in epithelial cells but it has less or no expression in normal liver cells.^125^, ^126^, ^127^, ^128^, ^129^ However, in the presence of adenovirus infection, HBV or cancer, the expression level of GP73 in the bile duct cells is not changed significantly, but it is consistently high in liver cells, which increases the expression of GP73. These results indicate that hepatocytes undergo some transformation when liver diseases occur, altering some of the factors that regulate the expression of GP73. Studying these regulatory mechanisms will thus help to understand pathogenesis of liver diseases.^130^, ^131^, ^132^, ^133^, ^134^, ^135^, ^136^

### 2.4 Influencing factors for GP73 expression

GP73 expression is affected by many factors, such as viral infections, for example, the GP73 expression is increased in adenovirus‐infected HepG2 cells. The GP73 protein and mRNA expression are also increased after adenovirus‐infected Hep3B liver cancer cells. The expression level of GP73 is no longer increased under lack of adenovirus E1A domain, indicating that the GP73 expression is associated with adenovirus infection.^137^, ^138^, ^139^, ^140^ The expression of GP73 is also increased in HBV‐replicated cells, whereas the expression of GP73 does not increase in cells transfected with HBV but without HBV replication, indicating that HBV replication may stimulate the expression of GP73, and hence, the GP73 expression is associated with HBV replication.^141^ Moreover, studies have shown that the GP73 expression levels are increased in HBV and HCV infections, giant cell hepatitis and alcoholic hepatitis. The expression level of GP73 is also increased in SK‐Hep‐1 cells fed with IFN‐γ, while it is decreased in cells added with IFN‐α. In addition, IL‐6 and IL‐1 can raise the GP73 expression, and TGF‐β can reduce the expression of GP73.^141^ Studies by Song et al showed that the Hsp90 inhibitor IPI‐504 can affect the expression of many tumour‐associated proteins and genes in pancreatic cancer. The amount of GP73 was found by to be decreased by immunohistochemistry, mass spectrometry and gene expression profiling.^142^, ^143^, ^144^

### 2.5 Functions of GP73

At present, the biological function of GP73 is not fully understood. The Golgi apparatus is a complex processing centre in the cell, and its abnormal function is closely related to occurrence and development of many diseases, such as muscular dystrophy and defects of innate glycoprotein glycosylation.^145^, ^146^ GP73 is located in the Golgi apparatus, so GP73 is very likely to be closely related to the structure and functions of Golgi apparatus, which are intracellular transport, protein modification and signal transduction. Studies have shown that the acidic and coiled‐coil domains in the C‐terminal domain are the main domains for GP73 protein expression; truncation of C‐terminal acidic domain of GP73 can lead to significant reduction of survival rates in mice.^147^ Various degrees of liver disease and kidney disease also be happened; for example, the GP73 C‐terminal coiled‐coil domain can bind sCLU to assist its post‐translational modification and delivery functions, and it can also bind apolipoprotein E to promote hepatitis C virus secretion.^148^, ^149^ The −2618 bp/−19 bp promoter at the GP73 gene level is closely related to biological function of GP73, and the GC box in its promoter can regulate the transactivation of adenovirus E1A, providing a basis for activation of adenovirus E1A. The core promoter contains a CpG island in the promoter region of housekeeping gene, suggesting that the GP73 may have functional part in the housekeeping genes.^51^ According to distribution of GP73, its phenotype and physiological functions are mainly concentrated in the regulation of digestive system, hippocampus and blood glucose. GP73 is lowly expressed in normal hepatocytes, and its high expression in liver diseases indicates that GP73 levels may be associated with liver stress conditions. Monitoring of GP73 expression in patients with liver disease may indicate liver abnormalities and can determine the patient's liver repair status and repair capacity.^150^

## 3 DETECTION METHODS FOR GP73

At present, the detection methods for biomarkers are attracting more and more attention,^151^, ^152^, ^153^ and the methods for examining the GP73 include Western blot, immunohistochemical staining and ELISA. Chen et al^154^ used Western blot to detect GP73 in the serum of healthy people and liver cancer patients, and they found that the sensitivity was 78% and specificity was 93.5% when incubated with polyclonal antibodies. The sensitivity when incubated with monoclonal antibodies was 84.7%, and specificity of 93.5%. The stability and reproducibility of monoclonal antibodies are better than that of polyclonal antibodies. However, due to complicated operation of Western blot, it is difficult to be widely used in clinical practice.

Yao et al^155^ used immunohistochemical staining for detection of GP73, and their results showed that the sensitivity was 75.4% and specificity was 82.8%. The sensitivity was lower than that of serological tests but the immune response from the diseased tissue could be visually observed. However, Western blot and immunohistochemistry methods are cumbersome, complicated, and their price is expensive, so they are not suitable for detection of large clinical samples. There is therefore still a need to find a simple, fast and inexpensive GP73 detection method suitable for clinical large sample detection.

Gu et al^156^ used ELISA to detect expression level of GP73 in the serum with 82% sensitivity and 80% specificity, and the ratio of GP73 in healthy people and liver disease patients was 1:3. However, there was no significant difference in GP73 levels in patients with hepatocellular carcinoma (HCC), cirrhosis and hepatitis, so the ELISA method could not meet the detection for liver cancer. Beijing Hotview Company invented a kit for the detection of serum GP73 using ELISA method. It has since been used in clinical practice in China. As a tool for judging liver fibrosis and cirrhosis, its sensitivity is 62.8% and its specificity is 80.5%.

In addition, nanomaterials have represented perfectly suitable materials for a variety of biomedical and biotechnological applications.^157^, ^158^, ^159^, ^160^, ^161^, ^162^, ^163^, ^164^, ^165^, ^166^, ^167^, ^168^, ^169^, ^170^, ^171^, ^172^ Our research group also established a variety of methodological detection methods. We used magnetic nanoparticle enzyme‐linked immunosorbent assay to detect serum GP73 levels in 79 cases of liver cancer and 64 healthy people. The sensitivity from our method was 78.43%, and specificity was 91.47%.^173^ In addition, the GP73 content in serum from 80 cases of liver cancer and 80 healthy people was detected by latex‐enhanced immunoturbidimetric method based on polyclonal antibody, with 94.6% sensitivity and 72.4% specificity. The use of three monoclonal antibody‐based latex‐enhanced immunoturbidimetric assays has a 96.7% sensitivity and 93.3% specificity.^2^ These two methods can be more easily automated, and they are faster than ELISA therefore have very good clinical application prospects.

## 4 RELATIONSHIP BETWEEN GP73 AND LIVER DISEASES

### 4.1 Relationship between GP73 and hepatitis

When liver cells develop autoimmune hepatitis or are infected with hepatitis B virus or hepatitis C virus, their GP73 expression levels will increase. When HBV and HCV are infected, the expression of GP73 in hepatocytes is increased significantly and do not change in the bile duct cells, suggesting that the active replication of hepatitis virus may be an important factor in the expression of GP73 in hepatocytes.^174^ Kladney et al^175^ found in 2000 that the GP73 expression increased during the progression of cirrhosis in autoimmune hepatitis, alcoholic liver disease, hepatitis B and liver disease. Iftikhar et al detected expression levels of GP73 in patients with autoimmune hepatitis, alcoholic liver disease, acute hepatitis and chronic hepatitis using Western blot, immunohistochemistry and immunofluorescence. They found that the GP73 levels increased in patients with liver disease, and GP73 levels in hepatocytes with alcoholic liver disease and chronic hepatitis were related to stage of the disease, regardless of the grade. Cells with alpha‐smooth muscle actin‐positive cells in liver sinusoidal endothelial cells also expressed GP73, suggesting that GP73 may be derived from activated hepatic stellate cells. In summary, they speculated that the secondary injury of chronic liver disease and the trigger mechanism for acute liver injury may lead to the expression of GP73.^140^ Wei et al found that the GP73 content in patients with chronic hepatitis C and HIV‐1 infection was significantly higher than that of healthy control group, and the increased GP73 was significantly correlated with body immune level and virus replication in HIV‐1‐infected patients. Therefore, patients with hepatitis C had too high GP73 and may not only be end‐stage liver disease or liver cancer and may also be combined with HIV‐1 infection, which also provides clinical clues for reference.^176^

### 4.2 Relationship between GP73 and cirrhosis

Liver cirrhosis is generally an end‐stage manifestation that occurs on the basis of diffuse liver damage, generally consisting of two mechanisms, namely, I: hepatocyte regeneration and proliferation of nodules; II: fibrous tissue proliferation and fibrous nodule formation, reconstruction of the original liver tissue and blood vessels. From hepatitis to liver fibrosis, liver cirrhosis and liver cancer are long and difficult to find process, and even if the alpha‐fetoprotein (AFP) detection abnormalities, but the imaging confirmation takes months or longer. Clinically, it is considered that 3 cm of HCC is an important demarcation point that is related to the prognosis. It takes at least 5 months for the tumour to grow from 1 cm to 3 cm, which means that the tumour can be detected after 5 months. The early diagnosis has important value for patient's treatment and prognosis.^92^ The study found that the expression level of GP73 in the liver cells increased significantly in patients with liver cirrhosis. Some scholars have studied 229 healthy people, liver cancer, liver cirrhosis and hepatitis patients and found GP73 expression levels in liver cancer and liver cirrhosis patients were significantly higher than in the healthy control group and hepatitis group.^34^ However, in another 535 studies, the GP73 expression levels in patients with cirrhosis were significantly higher than those in patients with liver cancer and hepatitis, and in the Child‐Pugh classification of cirrhosis, the GP73 levels were significantly higher in patients with grade B and grade C than those with grade A patients.^92^

Kladney et al used Western blot to analyse differences in GP73 content between cirrhosis and healthy subjects caused by different causes, including 17 healthy subjects, seven cases of autoimmune cirrhosis, nine cases of alcoholic cirrhosis and 14 patients with chronic hepatitis B cirrhosis and 23 patients with chronic hepatitis C cirrhosis. Their results found that the GP73 expression was elevated in patients with cirrhosis caused by different causes, but GP73 expression was not increased in healthy controls. The expression of GP73 in patients with cirrhosis caused by HBV was significantly increased by about 70 times. The level of GP73 in patients with cirrhosis, autoimmune cirrhosis and alcoholic cirrhosis caused by HCV was also elevated, but the level was not obvious. Their results suggested that the GP73 may play a crucial role in the pathogenesis of liver disease, but the specific mechanism is not yet clear.^177^

### 4.3 Relationship between GP73 and liver cancer

**Figure 2:**
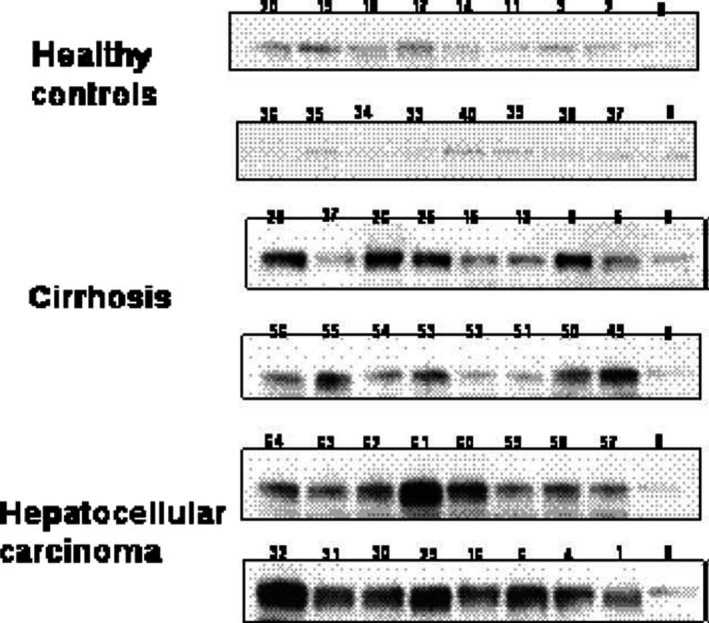
Immunoblot analysis of GP73 levels in controls, cirrhosis and hepatocellular carcinoma (HCC). The top panel shows immunoblot analyses of sera from controls without liver disease, the middle panel from cirrhosis and lower panel from subjects with HCC 184

At present, GP73 has attracted more and more attention as a serological marker for liver cancer.^178^ Most of the studies suggest that GP73 can improve the diagnostic rate for liver cancer (Table 1), and its sensitivity and specificity are higher than that of AFP, a traditional hepatoma serum marker. However, there is also controversy. Block et al used glycoproteomics to find that GP73 serum levels in American cornfields with HCC in 2005 were significantly higher than those without HCC, and GP73 levels in patients with HCC were significantly higher than those in patients with colon metastases and HBV infection.^183^ Marrero et al detected GP73 serum levels in 352 liver cancer patients, liver cirrhosis patients and healthy subjects using Western blot (Figure 2). Their results showed that GP73 serum levels in liver cancer patients were significantly higher than those in patients with liver cirrhosis. Compared with AFP, the area under the receiver operating characteristic (ROC) curve for GP73 was 0.79, which was significantly larger than the area under the ROC curve for AFP of 0.61. In the case of cut‐off for 10 times relative units, the sensitivity of GP73 was 69% and specificity was 75%, while the sensitivity for AFP was 30% and specificity was 96%. And when the AFP level was 100 and 20 ng/mL, the patients who exceeded the optimal cut‐off value were more than 71% and 62%, respectively, thus showing that the use of GP73 was more valuable in cases where the increased level of AFP is not significant or does not increase.^184^

**Table 1:** Detection methods and cut‐off values of GP73 in hepatocellular carcinoma (HCC) patients and controls

Methods	Patients with HCC/controls	Cut‐off values	References
Western blotting	13/93	7.4 relative units (RU)	180
Immunoblotting	102/179	8.5 RU	180
Immunoblotting	789/3428	8.5 RU	181
Immunoblotting	84/173	8.5 RU	104
ELISA	73/107	100 μg/L	29
ELISA	70/159	94.7 μg/L	182
ELISA	84/173	78.1 ng/L	4
Magnetic enzyme‐linked immunoassay	79/64	124.56 ng/mL	172
Monoclonal antibody‐based latex particle‐enhanced turbidimetric immunoassay	80/80	109.6 ng/mL	2
Polyclonal antibody‐based latex particle‐enhanced turbidimetric immunoassay	80/80	119.3 ng/mL	3

Hu et al^182^ studied the GP73 levels in 124 patients and found that the GP73 protein levels in patients with hepatitis‐related liver cancer were significantly higher than those in other liver diseases and healthy controls. The area under the ROC curve was 0.89, sensitivity was 77.4%, and specificity was 83.9%. After GP73 RNA was studied, the area under the ROC curve was 0.92, sensitivity was 87.1%, and specificity was 83.9%, indicating that the GP73 RNA detection was superior to GP73 protein detection. The detection of AFP revealed that the area under the ROC curve was 0.77, sensitivity was 48.4%, and specificity was 96.8%, indicating that the sensitivity for AFP detection was not as good as that of GP73.

**Figure 3:**
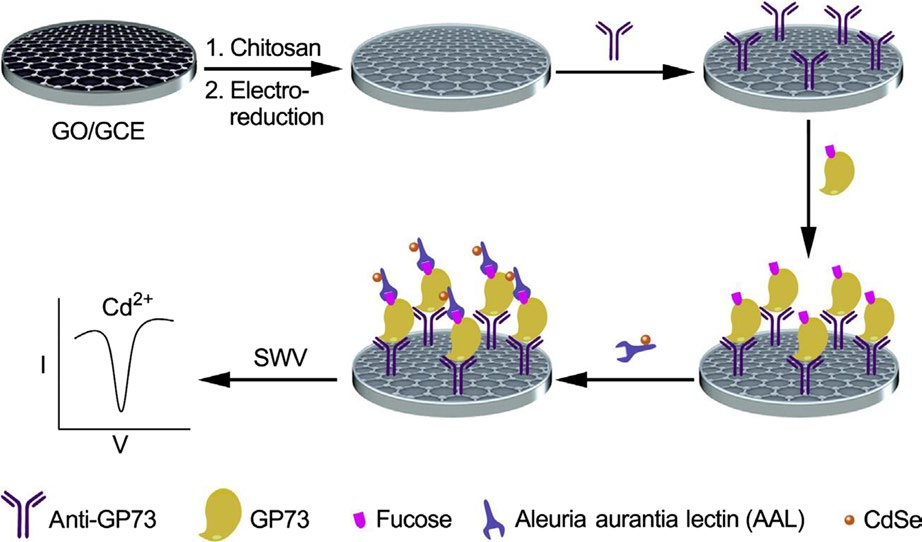
The proposed mechanism for BTC assay^185^

Yu et al^185^ developed an electrochemical immunosensor for assaying of Golgi Protein 73 (GP73), which is a newly established and highly sensitive biomarker for biliary tract cancer. Intrinsic specificity and sensitivity of this immunosensor can be achieved due to good conductivity of electrochemically reduced graphene oxide, and prominent electrochemical signal of quantum dots. So, our sensor can enable a limit of detection as low as 12 pmol/L, and it can make direct serum assay possible. Results from this assay may clearly predict the effect of surgical removal of primary tumour, indicating potential clinical application of our sensor in the future (Figure 3).

**Figure 4:**
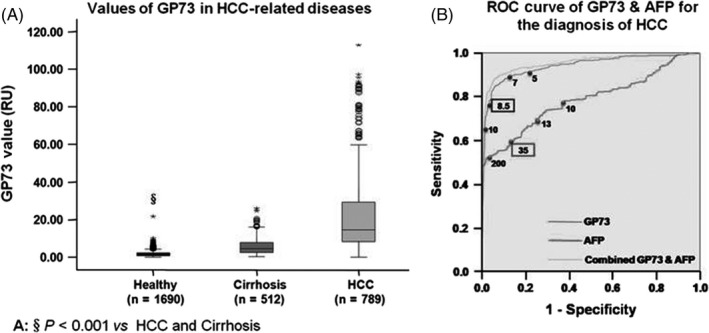
Serum Golgi protein 73 (GP73) is increased in patients with hepatocellular carcinoma (HCC), with higher sensitivity and specificity than alpha‐fetoprotein (AFP). A, The GP73 values for HCC‐related diseases. The serum GP73 level for healthy subjects, patients with cirrhosis and patients with HCC was 1.2 (0.9e1.7) relative units (RU), 4.7 (2.6e8.0) RU and 14.7 (8.4e29.4) RU, respectively. ^§^ *P* < 0.001 vs HCC and cirrhosis. B, The receiver operating characteristic (ROC) curve for combined AFP and GP73 in HCC diagnosis of all subjects. The points in the ROC curve indicate different GP73 values with corresponding sensitivity and specificity, from which the cut‐off value was chosen, and 8.5 RU (boxed) was the cut‐off value for GP73^186^

Mao et al^186^ investigated the relationships between GP73 levels and tumour size or differentiation status or liver function. For comparison, AFP values for different tumour sizes or differentiation status or liver function were also analysed. The GP73 values were not correlated with tumour size, histological differentiation or Childe‐Pugh class. In contrast, the AFP values for patients with small HCCs (≤3 cm) were significantly less than that of other HCCs (≥5 cm, >3 and <5 cm, and diffuse HCC) (*P* < 0.001). GP73 was positive (≥8.5 relative units) in 30 of 34 (88.4%) AFP‐negative (<35 ng/mL) patients with small HCCs, while AFP was positive in three of seven (42.9%) GP73‐negative patients with small HCCs (Table 2). The sensitivity of GP73 for detection of liver cancer was 74.6%, and specificity was 97.4%, while the sensitivity of AFP for detection of liver cancer was 58.2% and specificity was 85.35%. This indicated that the GP73 was superior than AFP in detecting liver cancer. However, the combined diagnosis of GP73 and AFP is more likely to increase the detection rate of liver cancer (Figure 4). The diagnostic accuracy of serum GP73 and AFP in the same patient population was assessed in Table 3. The results demonstrated that GP73 showed higher sensitivity than AFP for HCC diagnosis. However, several studies showed the specificity of GP73 was lower than AFP, which might be due to that GP73 can be expressed in many types of cells, but the maior cell type was epithelial cells.

**Table 2:** GP73 and alpha‐fetoprotein (AFP) of hepatocellular carcinoma (HCC) with different tumour sizes, differentiations and liver functions^186^

Tumour size (cm)	≤3 (n = 45)	>3 and <5 (n = 126)	≥5 (n = 197)	Diffuse (n = 55)
GP73 value (RU)	16.1 (10.6‐42.6)	14.1 (6.8‐42.6)	18.6 (12.3‐38.8)	17.6 (8.3‐35.1)
AFP (ng/mL)	29.8 (10.1‐34.9)[Link]	102.7 (11.2‐360.4)	129.6 (10.8‐353.2)	121.8 (11.0‐358.7)

Tumour differentiation	High (n = 52)	Moderate (n = 80)	Low (n = 48)
GP73 value (RU)	12.9 (7.4‐46.2)	19.0 (8.3‐39.3)	17.6 (9.9‐65.2)
AFP (ng/mL)	119.0 (20.8‐478.6)	129.8 (18.9‐442.5)	143.1 (12.9‐465.3)

Childe‐Pugh class	A (n = 221)	B (n = 162)	C (n = 43)
GP73 value (RU)	15.6 (10.2‐38.9)	14.6 (9.4‐41.2)	16.8 (11.0‐52.6)
AFP (ng/mL)	108.9 (14.0‐395.6)	122.6 (24.8‐410.2)	124.6 (22.1‐469.3)

*P* < 0.001 vs >3 and <5, ≥5 and diffuse HCC.

**Table 3:** Comparison of sensitivity and specificity of alpha‐fetoprotein (AFP) and GP73 in hepatocellular carcinoma

Authors	No. of patients	AFP				GP73			
		Detection method	Cut‐off value	Sensitivity (%)	Specificity (%)	Detection method	Cut‐off value	Sensitivity (%)	Specificity (%)
Marrero et al^184^	352	Immunoassays utilizing enhanced chemiluminescence	112 ng/mL	25	97	Immunoblotting	10 relative units	62	88
Mao et al^186^	4217	Immunoassays utilizing enhanced chemiluminescence	35 ng/mL	58.2	85.3	Immunoblotting	8.5 relative units	74.6	97.4
Sai et al^43^	328	Immunoassays utilizing enhanced chemiluminescence	50 μg/L	71.97	84.48	ELISA	70 μg/L	78.34	77.59
Wang et al^40^	257	Immunoassays	13.5 relative units	60.7	75	Immunoblotting	13.5 relative units	82.1	80
Hou et al^77^	184	Chemiluminescence immunoassay	—	55.6	86.7	Time‐resolved fluorescence immunological assay	78.1 ng/L	73.4	79
Hu et al^182^	124	Electrochemiluminescence immunoassay	—	48.4	96.8	Western blotting	7.4 relative units	77.4	83.9

Although GP73 has great potential as a diagnostic marker for liver cancer, there are still many differences. For example, most studies reported that the serum levels of GP73 in patients with liver cancer were higher than those in other liver diseases, but there were also some opposite results. Tian et al^187^ used ELISA to detect 535 samples, including liver metastatic adenocarcinoma, intrahepatic cholangiocarcinoma, hemangioma, focal hyperplasia, hepatitis, liver cirrhosis, liver cancer and healthy people. Their results showed that GP73 levels in cirrhotic patients were higher than those in patients with liver cancer and hepatitis. Gu et al^156^ detected serum GP73 by ELISA. Their results showed that the GP73 content of liver disease patients was higher than that of healthy control group, but there was no difference in the liver disease patients from each group. These differences may be related to individual differences in the subjects, sample collection, preservation differences, differences in reagent use, differences in detection methods and so on. Therefore, obtaining samples from different populations in different regions in the study, expanding the number of test samples and establishing more effective and reliable detection methods are of great value for accurate GP73 detection in liver cancer.

Besides, exosomes are 30‐100 nm secreted vesicles that have emerged as a novel mode of diagnostic marker and demonstrated to play important roles in cellular intercommunication. They are enriched in various bioactive materials, including proteins, lipids, miRNAs and mRNAs.^188^ Exosomes have also been found in several types of body fluids, including plasma, malignant effusions, urine, saliva and amniotic fluid.^189^ In addition to some common exosomal proteins such as TSG101, Alix, CD9, CD81, CD63 and cytoskeletal proteins including actin, and tubulin proteins, exosomes may carry some disease‐associated proteins for they are a sub‐part of originated cells.^190^ Several researches have shown that the GP73 protein is present in exosome surface.^191^, ^192^, ^193^, ^194^ The advantages of exosomes as tumour markers are as follows: (a) a large quantity, the concentration of exosomes in the serum can reach 3 × 10^6^ cells/μL.^195^ On average, each tumour cell can release 530 exosomes/24 hour^196^; (b) information rich and similar to tumour cells; the surface of exosomal membrane also has the same protein as cell membrane, which can represent characteristics and state of its parental cell^197^; (c) it is easy to preserve for a long time and remove interference; there is phospholipid bilayer to protect its degradation by proteases and nucleases, and as long as the separation of exosomes can remove the interference^198^; and (d) non‐invasive detection; exosomes can enter the circulatory system through endothelium and can therefore be detected in almost all body fluids.^199^ Compared to tissue biopsy, exosomes are effective biomarkers for personalized medicine. Therefore, analysis of GP73 protein in exosome surface will be valued in the diagnosis of liver cancer.

## 5 CHALLENGES AND STRATEGIES

In summary, the serum levels of GP73 in patients with liver diseases, such as hepatitis, liver cirrhosis and liver cancer, have increased in varying degrees. Although accumulating studies indicate that abnormal GP73 expression is associated with tumour progression by interacting with the microenvironment, and GP73 has been regarded as a potential diagnostic marker for HCC, the diagnosis accuracy of GP73 in cirrhosis and HCC distinguishment is worthy discussed.^20^, ^22^, ^187^ Although some studies found that ELISA result did not show a significant elevation of serum GP73 in HCC groups compared with that in liver cirrhosis groups. Recent reports described that GP73‐specific serum autoantibodies might interfere with ELISA analysis. Researchers have found several isoforms of GP73 that correspond with different patterns or levels of glycosylation.^200^, ^201^, ^202^ It still needs further research about the significance of HCC‐specific GP73 isoform in diagnostic accuracy improvement. Moreover, GP73 tends to be used as a useful biomarker to monitor HCC prognosis after primary tumour surgery and therapy in advanced HCC.^132^, ^133^ In terms of clinical application, it is still a new biomarker, and large‐scale and multi‐centred investigations are still needed. Moreover, more sensitive, specific and rapid diagnostic methods are also needed. Since exosomes have many advantages, and a new detection method for GP73 from the perspective of exosomes is imperative to enhance the role of GP73 in human health research.
